# Trends of accidental carbon monoxide poisoning in Korea, 1951-2018

**DOI:** 10.4178/epih.e2020062

**Published:** 2020-08-31

**Authors:** Jong-Hun Kim, Ah-Young Lim, Hae-Kwan Cheong

**Affiliations:** Department of Social and Preventive Medicine, Sungkyunkwan University School of Medicine, Suwon, Korea

**Keywords:** Carbon monoxide poisoning, Coal, Briquette gas poisoning, Time trend, Mortality, Morbidity

## Abstract

**OBJECTIVES:**

Carbon monoxide (CO) poisoning from coal briquette combustion has been a major public health problem in Korea. In this study, we estimated the time trends of the consumption of anthracite coal and the number of CO poisoning victims over the past 7 decades, in the context of changes in heating facilities.

**METHODS:**

Using Population and Housing Census data and energy statistics, we estimated the number of houses using briquettes as heating fuel between 1951 and 2018. After estimating the incidence of CO poisoning in housing units by heating facility type, we determined the ratio of the number of household members who experienced CO poisoning to the overall number of household members. Finally, we estimated the distribution of the victims according to poisoning severity, excluding victims of intentional exposure.

**RESULTS:**

We estimated that, overall, over 26 million people experienced CO poisoning between 1951 and 2018 in Korea. The household consumption of anthracite peaked in 1986, but the number of victims of CO poisoning peaked at approximately 1 million people in 1980. From 1951 to 2018, the cumulative number of CO poisoning victims comprised approximately 22,830,000 mild cases, 3,570,000 severe cases, and 65,000 deaths.

**CONCLUSIONS:**

The peak in the number of CO poisoning victims occurred 6 years earlier than the peak in the number of people using briquettes for heating. This gap resulted from improvements in briquette heating systems. This finding provides a quantitative basis for epidemiological studies on the health outcomes of CO poisoning in the Korean population.

## INTRODUCTION

Carbon monoxide (CO) poisoning, called “the silent killer,” is caused by inhaling CO, a colorless, odorless gas formed from the incomplete combustion of coal and other hydrocarbon products [1]. CO binds more strongly to hemoglobin in the blood than does oxygen, hindering the supply of oxygen to the tissues [2]. CO poisoning causes symptoms such as headaches, dizziness, nausea, and vomiting. In severe cases, it may cause unconsciousness or even death [3].

Before the 1960s, traditional residences in Korea used wood as a main fuel for heating and cooking. As the government began to regulate logging in the mountains to protect forests that were destroyed during the Korean War, coal replaced firewood in the private sector [4-6]. In the 1960s, coal briquettes began to be used as house fuel in Korea, and CO poisoning became a serious social and public health issue [4]. The problem of CO poisoning, which has lasted for more than 40 years, has gradually become less severe since the 1990s, when home heating and cooking fuels were replaced with petroleum and liquefied natural gas (LNG). Korean traditional house heating uses the *ondol* heating method, in which hot smoke warms the floor of a room [7]. Due to the structural characteristics of *ondol* rooms, which inevitably have small leaks, gases from combustion could seep through the gaps in the floor [8]. According to a survey of the leading causes of accidental CO poisoning between 1965 and 1976, gas leaking from *ondol* structures accounted for 54.2% of accidents, while gas leaking from a fireplace such as a coal fuel hole accounted for 26.3%. Overall, about 80% of CO poisoning cases could be attributed to *ondol* heating facilities [9]. By that time, CO poisoning had become a major social problem due to the combination of traditional heating methods and poor residential housing created by rapid urbanization. According to police data from this time, 85% of CO poisoning cases occurred in slums on the outskirts of the city [4]. Not only did the slums have poor housing conditions, but the residents were particularly vulnerable to CO poisoning as a result of the high prevalence of anemia due to malnutrition [4].

Epidemiological studies have been conducted on CO poisoning since the 1980s in parallel with the widespread availability of hyperbaric oxygen tanks. However, most of these studies were based on limited data sources or direct surveys conducted in metropolitan areas. In the past, data on deaths from CO poisoning were often missing. Registry statistics were maintained by the police. However, patients with CO poisoning who received hospital treatment were not managed by the public health sector [9]. Therefore, the nationwide number of victims of CO poisoning has not been systematically investigated. Since the 2000s, briquette usage has drastically decreased, and the number of victims of CO poisoning has also steadily declined, while coal briquettes were still used among the energy-poor class in Korean. Moreover, studies of the extent of health damage due to CO poisoning have been insufficient. Consequently, no estimation is available of the incidence of CO poisoning across the overall period of briquette use in Korean society.

Assessing the overall scale of CO poisoning provides an estimate of the incidences of mild, severe, and fatal cases. Especially in severe cases, CO poisoning can have neurological sequelae, which are known to last for life. Therefore, understanding the scale of past CO poisoning in present viable age groups can provide an essential data source for follow-up studies related to such sequelae. The purpose of this study was to investigate the amount of briquette consumption and estimate the incidence of CO poisoning in Korea between 1951 and 2018.

## MATERIALS AND METHODS

In the 1960s and 1970s, insufficient data were available to estimate the health effects of CO poisoning. In the 1980s and 1990s, cause-of-death data existed, but many cases were missing, and errors were often included because the death registration system was not yet systematic and complete. Therefore, we performed the following procedure to estimate the health consequences of CO poisoning from the use of briquettes. First, Population and Housing Census data and the yearbook of energy statistics were used to determine the scale of use of briquettes as heating fuel over a long time period. Next, to estimate the incidence of the health outcomes of CO poisoning, previous epidemiological studies were reviewed.

### Data sources

A questionnaire on household heating fuel was conducted every 5 years to 10 years (in 1960, 1970, 1975, 1980, 1985, 1990, 2000, 2005, 2010, and 2015) as part of the Population and Housing Census [10]. From the census data, we acquired information on variables such as the numbers of houses using briquettes as heating fuel and the number of persons per household (Table 1). The Population and Housing Census data used in this study represent a 2% sample. Due to the complexity of the data, a stratified sampling method was applied according to the type of residence by administrative district and the number of household members, and weights were applied for post-stratification. In the estimation of the number of houses using briquettes for heating, the confidence interval was calculated by applying the relative standard error and design factors. More detail about the calculation methods used can be found in the Population and Housing Census user guide. For the intervening years without census data, we extrapolated from the data for the nearest preceding and subsequent years when the census was conducted. In Korea, heating fuel use patterns changed meaningfully between 1990 and 2000. However, Population and Housing Census data exist only for 1990 and 2000, making it challenging to identify fluctuations during the intervening 10 years. To solve this problem, we acquired data on the number of houses using briquettes from the yearbook of energy statistics provided by the Korea Energy Statistical Information System (Supplementary Material 1). These data were used to supplement the data on the number of people using briquettes from 1990 to 2000 [11]. To compare the consumption of briquettes and the number of victims from CO poisoning, we used the anthracite demand in the residential and commercial domains obtained from the 1951-2018 data provided by the Korea Coal Association [12]. Data on the incidence of CO poisoning from heating systems and the distribution of victims according to the severity of CO poisoning were obtained from previous epidemiological studies [13,14]. To verify the validity of the estimated number of deaths from CO poisoning, we compared our findings with the police statistical yearbook and annual cause-of-death statistics based on the International Statistical Classification of Diseases and Related Health Problems code published by the National Statistical Office [15] (Supplementary Material 2).

### Statistical analysis

**Figure 1.**
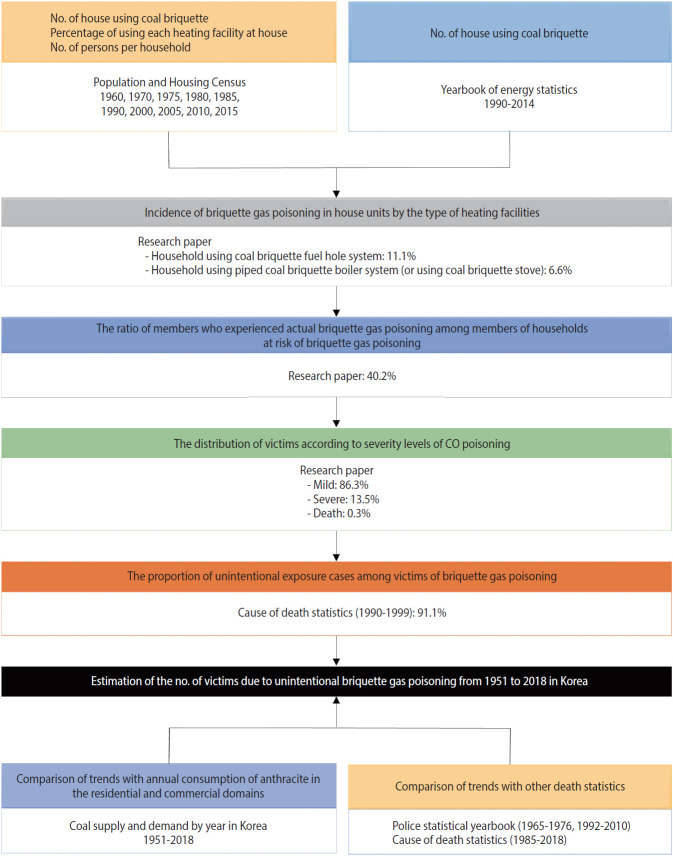
Flowchart of the estimation of the number of victims of carbon monoxide (CO) poisoning.

The procedure for estimating the number of victims of accidental CO poisoning in Korea was as follows. First, data from the Population and Housing Census and the yearbook of energy statistics were used to estimate the number of houses using coal briquettes as heating fuel between 1951 and 2018 (Figure 1). Second, the total number of houses at risk was calculated by applying the incidence of CO poisoning in housing units based on the type of heating facility to the number of houses that used briquettes as heating fuel. Third, among the houses at risk of CO poisoning, we calculated the ratio of the number of members who experienced CO poisoning to the total number of household members. Fourth, we used the distribution of victims by the severity level of CO poisoning to estimate the number of victims of mild, severe, and fatal cases in each year. Fifth, since CO poisoning cases include cases of intentional exposure for suicide, we excluded the proportion of CO poisoning due to intentional exposure and calculated only the number of victims of unintentional briquette gas exposure. Finally, we compared the amounts of annual anthracite consumption in residential and commercial domains to the trends in the number of victims of unintentional CO poisoning. We also validated our results by comparing them with statistical police yearbook data and cause-of-death statistics.

### Ethics statement

Since individual subjects were not enrolled, there were no ethical concerns to be clarified. Ethical clearance was neither applicable.

## RESULTS

In 1960, the proportion of houses using briquettes as heating fuel was 24.1%, and this number continued to rise to a peak of 69.5% in 1980. In 1985, the proportion of houses using briquettes as a heating fuel was still high (67.7%), but a significant change in heating systems had occurred. In 1980, the old system, involving a coal briquette fuel hole, represented approximately 50% of heating systems, but by 1985, the improved pipe briquette boiler system represented approximately 50% of heating systems. Since 1990, the proportion of houses using briquettes has decreased to 62.0% due to increases in the usage of other heating fuels. In addition, between 1990 and 2000, a drastic change in heating systems occurred. As a result, the proportion of houses using briquettes as a heating fuel decreased to only 1.8% by 2000 (Table 1).

**Figure 2.**
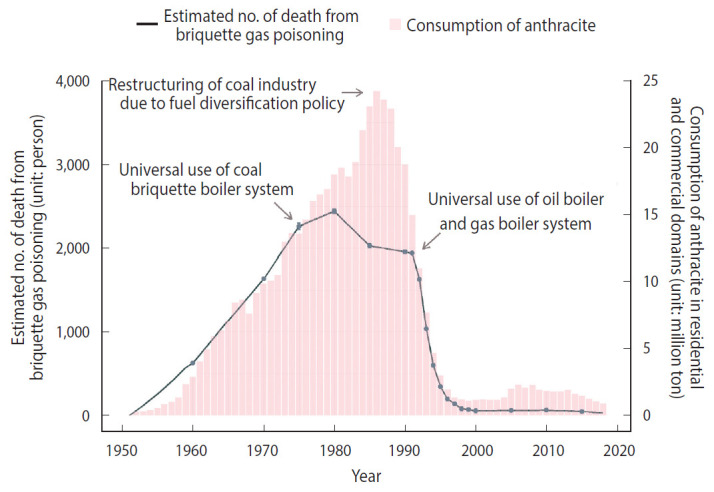
Estimated number of victims from carbon monoxide poisoning and anthracite consumption in Korea, 1951-2018.

We estimated the number of people using briquettes, calculated by multiplying the number of houses using briquettes by the number of members per household, as 17,214 in 1951 (Supplementary Material 3). Subsequently, the number of people using briquettes steadily increased to over 1 million in 1953 and over 10 million in 1965. It exceeded 20 million in 1974 and reached a peak of approximately 27 million in 1988. These results were similar in pattern to the consumption of civilian anthracite coal, which peaked in 1986 (Figure 2).

The trend in the number of people experiencing CO poisoning differed somewhat from the pattern of civilian anthracite coal consumption and that of the number of people using briquettes (Figure 2). The estimated number of people who experienced CO poisoning peaked at approximately 1 million in 1980. This peak occurred approximately 6 years to 8 years earlier than the peak in the number of people using briquettes as heating fuel and the peak in the consumption of civilian briquettes.

The numbers of mild, severe, and fatal cases followed the same trend, as the proportions were based on the population estimated to be at risk of CO poisoning. The number of mild cases peaked in 1980 at approximately 0.86 million cases, while severe cases numbered approximately 134,000 that year. The number of fatal cases first exceeded 1,000 in 1964, and it was estimated that at least 1,000 annual deaths continued during the subsequent 30 years, until 1993. The number of fatal cases peaked in 1980, with approximately 2,400 deaths (Supplementary Material 3).

## DISCUSSION

In Korea, briquette use became widespread following the Korean War in the 1950s [5,6]. As a result, 24.1% of all houses used briquettes as a heating fuel according to the 1960 Population and Housing Census. In 1988, the number of people using briquettes as a heating fuel peaked, and since then, it has gradually declined due to the proliferation of other heating systems. In the 1990s, in particular, the heating fuels of choice rapidly changed to oil and LNG due to increased apartment availability and a change in government energy policy.

The incidence of CO poisoning has fluctuated with the changes in heating facilities and subsequent improvements to residential environments and with changes in the number of briquettes consumed per day. In particular, although briquettes were used as a heating fuel in most houses in the 1980s, the incidence of CO poisoning differed according to the details of the heating facility. Among facilities using the briquette fuel hole system, the annual probability of CO poisoning was 11.1%, while the more advanced piped-coal briquette boiler system was associated with a probability of 6.6% [13]. The briquette fuel hole system, which has been in use since the 1960s, is particularly vulnerable to briquette gas leaks because it relies on combusted hot air passing underneath an *ondol* floor. By contrast, the piped-coal briquette boiler system, which has been in use since the mid-1970s, is a hot water radiant floor heating system. Therefore, the thermal energy efficiency of the piped-coal briquette boiler system is high, and the risk of briquette gas leakage is relatively low compared with the fuel hole system [7]. In houses using briquette fuel hole systems, people using briquettes from evening to morning tended to use fewer air holes to extend the length of briquette usability [5]. This increased the amount of incomplete combustion, leading to increased CO generation and a rise in the incidence of CO poisoning. Based on the duration of usage for a single-use briquette, the briquettes were likely replaced 2-3 times a day. In most Korean houses, briquettes were generally replaced just before sleeping, shortly after waking, and in the afternoon [16]. Since CO concentrations reach their peak levels 2-3 hours after starting to burn a briquette, many people who changed to new briquettes before going to sleep were at risk for CO poisoning during deep sleep [9]. For the reasons already discussed, although annual anthracite consumption in the residential and commercial domains peaked in 1986, cases of briquette gas poisoning occurred most frequently in the late 1970s and early 1980s, even though briquette usage in this period was lower than in the mid-1980s.

The symptoms of CO poisoning differ in severity depending on the concentration of inhaled CO and the exposure time. Therefore, to estimate the number of victims of CO poisoning, measuring the incidence by severity is required. CO poisoning symptoms can be classified as mild, severe, or fatal. In this study, cases involving headache, nausea, and vomiting were classified as mild, and cases involving temporary loss of consciousness were classified as severe. In the past, national statistical surveys on CO poisoning cases were not conducted in Korea, so large-scale surveys of more than 100,000 people have only been conducted by individual researchers. In the 1970s and 1980s, 2 population surveys of more than 100,000 people regarding their CO poisoning experiences in Korea were published (Table 2). In a 1974 survey of approximately 530,000 people living in Seoul, for every 10,000 people using briquettes as heating fuel, 244 people experienced mild symptoms, 45 suffered severe symptoms such as loss of consciousness, and 1 individual died as a result of CO poisoning [17]. In addition, a study conducted in 1984 estimated that annually, for every 10,000 people using briquettes, 320 people suffered mild symptoms, 50 people experienced severe symptoms, and 1 person died from CO poisoning [14].

In Korea, causes of death were first officially classified in 1979. However, the cause-of-death statistics compiled in the 1980s and 1990s had many problems [18]. In 1980, only 45% of deaths were classified by cause, and only 24% of registered deaths were certified by doctors [19]. The proportion of death certificates written by doctors remained low at 36%, 57%, and 60% in 1985, 1994, and 1997, respectively. Additionally, the cause-of-death data contained many errors [18-20]. Surveys in 1991 found errors in the cause of death in 56.1% of all cases [20]. Also, continuous reports were made of missing or distorted death registries until the mid-1990s. For example, in the case of suicide deaths, a difference of approximately 3,000 people was present between the police statistics and the cause-of-death statistics [20]. For these reasons, it can be concluded that a portion of the deaths due to CO poisoning in the 1980s and early 1990s may have been misclassified as death from unknown causes. Therefore, it is likely that the official annual cause-of-death statistics on CO poisoning were underestimated. Nevertheless, in 1985, approximately 1,600 people had a cause of death classified as ‘accidental poisoning by gas and steam’ [21]. These findings helped us interpret and explain our results regarding the estimated number of deaths from CO poisoning in Korea over the past 70 years. From 1985 to 1999, our estimates were approximately 2 times to 3 times greater than the official data of the National Statistical Office. Based on these results, we estimate that many deaths from CO poisoning were missing or misclassified in the official death registry data prior to the 2000s. These shortcomings began to be addressed in the 2000s. According to one study, reported causes of death were at least 90% accurate beginning in the 2000s [22]. In our study, the estimated number of deaths from CO poisoning since 2000 was similar to that from the official death registry.

In the past, CO poisoning was classified as an accident, so it was not reported separately from accidents. Statistics produced by the police included only patients who were found and identified as dead at the site of CO poisoning, meaning that patients who died during hospital treatment were not included in the statistics [18]. According to the police statistical yearbook of 1976, 1,013 people died from CO poisoning that year [9]. However, according to a study conducted in the 1980s, the number of people who died due to worsening symptoms while being treated for CO poisoning at a hospital was 1.6-fold higher than the number of those who died at the scene [13]. Therefore, the number of deaths from CO poisoning reported by the National Police Agency in 1976 was likely underestimated [8]. Based on the factors leading to these underestimated statistics, we estimate that the number of annual deaths from CO poisoning in the mid-1970s may have exceeded 2,000. By combining sources of data from the past, we can confirm our estimations of causes of death, which were inferred based on the number of people who used briquettes as heating fuel.

Reports of cases of CO poisoning typically combine the number of cases of intentional exposure for suicide and the number of cases of unintentional poisoning caused by accidental exposure. Therefore, we used epidemiological data in the form of cause-of-death statistics to distinguish these 2 situations. Before 2007, most CO poisoning deaths in Korea were unintentional poisoning deaths caused by exposure to briquettes, and intentional poisoning deaths were rare [23]. The death registry from 1990 to 1999 shows that the proportion of suicide in cases of CO poisoning was about 8.9% (Supplementary Material 4). Because these figures were nearly constant, we used them to estimate the number of deaths from accidental CO poisoning.

The limitations of this study are as follows. First, in Korea, very few epidemiological papers have been published on the incidence of CO poisoning. In this study, we applied the average value of the incidence rates calculated in 4 Korean cities. However, since our analyses were performed only for a single year, our results may not accurately reflect the incidence of CO poisoning because of improvements in housing and changes in heating facilities. Second, in estimating the number of houses using briquettes, we used data from the Population and Housing Census. However, since the Census was conducted at 5-year or 10-year intervals, data for the remaining years had to be extrapolated, which can introduce instability to the estimates. Third, since the 2000s, the use of LNG has spread, and cases of CO poisoning from gas boiler exhaust emissions have occurred. In addition, the use of instantaneous gas water heaters has caused accidents resulting from incomplete combustion. As outdoor camping has gained popularity, CO poisoning from associated heating devices has begun to occur while people sleep. Thus, it is challenging to assert that our methodology completely accounts for all cases of CO poisoning since the 2000s; however, since the 2000s, the number of people using briquettes as a heating fuel has declined significantly, so this issue is unlikely to introduce any significant errors. Finally, this study did not account for the effects of hyperbaric oxygen therapy, which has been the main therapeutic option for CO poisoning in Korea since the 1970s, but limited longitudinal data are available on its usage. A consensus exists on the effect of hyperbaric oxygen therapy in reducing the neurological sequelae of CO poisoning, but its effect on mortality still needs to be established [25]. Despite these limitations, this study describes trends in the number of victims of CO poisoning after exposure to briquette gas over the past 70 years. Similar findings have been reported in several epidemiological studies on the incidence of CO poisoning in Korea, so these results can be considered very accurate. Additionally, the data from the Population and Housing Census of Korea are highly reliable, as this census was applied to the entire nation.

In this study, we identified changes in heating systems and annual coal consumption in Korea over the past 70 years. Based on these data, we estimated the number of people using briquettes as a heating fuel during this time period. In addition, we calculated the number of victims and thus the incidence of CO poisoning, as well as the severity of illness by type of heating facility. We confirmed that the peak number of CO poisoning victims occurred about 6 years earlier than the peak in annual briquette consumption. We estimated that over 17 million people experienced CO poisoning in the 1970s and 1980s and that approximately 100,000 people were severely affected each year. We also estimated that more than 2,000 people died annually at the peak of CO poisoning. Severe CO poisoning is known to cause neuropsychiatric sequelae such as cerebral palsy by inducing brain hypoxia [5,24]. Since these effects are persistent in CO poisoning survivors, the health effects of the high rates of CO poisoning in the 1970s and 1980s are likely to be ongoing. Therefore, estimating the size of the Korean population that suffered from CO poisoning in the past is helpful for understanding the disease burden due to the neurological sequelae caused by CO poisoning in the present living population. In addition, since coal briquettes are still widely used as a heating fuel in North Korea, the results of this study could be used as an important data source for estimating the extent of CO poisoning in the North Korean population in the future.

## Figures and Tables

**Table 1.** Estimated number of households using briquettes as heating fuel, based on Population and Housing Census data

Year	No. of houses	Households using briquettes for heating		
		Direct briquette-heated system (coal briquette fuel hole system)	Heat-piped briquette boiler system (piped-coal briquette boiler system)	Coal briquette stove
1960	4,370,599	1,000,905 (867,842, 1,133,969)	-	52,483 (17,991, 86,975)
		22.9 [18.9, 26.0]	-	1.2 [0.4, 2.0]
1970	5,792,767	2,628,709 (2,553,023, 2,704,396)	238,096 (207,915, 268,277)	150,906 (126,691, 175,121)
		45.4 [44.1, 46.7]	4.1 [3.6, 4.6]	2.6 [2.2, 3.0]
1975	6,750,350	3,550,414 (3,248,396, 3,852,432)	702,950 (518,206, 887,693)	223,856 (115,551, 332,161)
		52.6 [48.1, 57.1]	10.4 [7.7, 13.2]	3.3 [1.7, 4.9]
1980	7,969,201	3,968,304 (3,759,700, 4,176,908)	1,291,344 (1,137,606, 1,445,082)	278,897 (202,225, 355,569)
		49.8 [47.2, 52.4]	16.2 [14.3, 18.1]	3.5 [2.5, 4.5]
1985	9,571,361	1,667,089 (1,454,900, 1,879,278)	4,816,026 (4,536,291, 5,095,761)	-
		17.4 [15.2, 19.6]	50.3 [47.4, 53.2]	
1990	11,354,540	1,555,560 (1,385,249, 1,725,872)	5,485,427 (5,237,911, 5,732,942)	-
		13.7 [12.2, 15.2]	48.3 [46.1, 50.5]	
2000	14,310,126	45,192 (16,319, 74,065)	217,434 (154,485, 280,383)	-
		0.3 [0.1, 0.5]	1.5 [1.1, 2.0]	
2005	15,887,128	48,032 (18,254, 77,811)	245,432 (178,539, 312,325)	-
		0.3 [0.1, 0.5]	1.5 [1.1, 2.0]	
2010	17,341,966	27,091 (5,099, 49,083)	323,202 (247,894, 398,510)	-
		0.2 [0.0, 0.3]	1.9 [1.4, 2.3]	
2015	19,111,731	24,101 (3,028, 45,174)	267,162 (197,450, 336,874)	-
		0.1 [0.0, 0.2]	1.4 [1.0, 1.8]	

Values are presented as estimated number (95% confidence interval) and % [95% confidence interval].

**Table 2.** Incidence per 10,000 people at risk for briquette gas exposure and distribution of victims according to the degree of carbon monoxide (CO) poisoning in previous studies

Degree of CO poisoning	Incidence per 10,000 population at risk of briquette gas exposure (the distribution of victims)	
	A study on CO poisoning in 1974 (study area: Seoul) [17]	A study on CO poisoning in 1984 (study area: Seoul, Busan, Daegu, and Daejeon) [14]
Mild	244 (79.8)	320 (86.3)
Severe	45 (14.5)	50 (13.5)
Fatal	1 (0.3)	1 (0.3)
Unspecified	16 (5.4)	0 (0.0)
Total	306 (100)	371 (100)

Values are presented as number (%).
