# Supplementary material for: Trends of accidental carbon monoxide poisoning in Korea, 1951-2018
Source: Epidemiol Health. 2020 Aug 31;42:e2020062. doi: 10.4178/epih.e2020062 (PMC7871165; doi:10.4178/epih.e2020062)
Supplement: Supplementary Material 1. [file epih-42-e2020062-suppl1.docx]

Supplementary Material 1. Number of households using briquettes as a heating fuel based on yearbook energy statistics

(Unit: 1,000 house)

| Year | Total houses | Number of houses using briquettes as heating fuel | (%) |
| --- | --- | --- | --- |
| 1990 | 11,355 | 7,180 | 63.2 |
| 1991 | 11,272 | 7,144 | 63.4 |
| 1992 | 11,755 | 6,162 | 52.4 |
| 1993 | 12,186 | 3,997 | 32.8 |
| 1994 | 13,103 | 2,379 | 18.2 |
| 1995 | 12,958 | 1,412 | 10.9 |
| 1996 | 14,242 | 832 | 5.8 |
| 1997 | 14,844 | 588 | 4.0 |
| 1998 | 14,852 | 353 | 2.4 |
| 1999 | 15,115 | 316 | 2.1 |
| 2000 | 14,391 | 241 | 1.7 |
| 2001 | 14,834 | 223 | 1.5 |
| 2002 | 15,064 | 191 | 1.3 |
| 2003 | 15,298 | 157 | 1.0 |
| 2004 | 15,539 | 185 | 1.2 |
| 2005 | 15,988 | 201 | 1.3 |
| 2006 | 17,488 | 208 | 1.2 |
| 2007 | 16,417 | 287 | 1.7 |
| 2008 | 16,673 | 204 | 1.2 |
| 2009 | 16,917 | 174 | 1.0 |
| 2010 | 17,152 | 159 | 0.9 |
| 2011 | 17,380 | 146 | 0.8 |
| 2012 | 18,057 | 130 | 0.7 |
| 2013 | 18,206 | 121 | 0.7 |
| 2014 | 18,458 | 97 | 0.5 |
