# Supplementary material for: Trends of accidental carbon monoxide poisoning in Korea, 1951-2018
Source: Epidemiol Health. 2020 Aug 31;42:e2020062. doi: 10.4178/epih.e2020062 (PMC7871165; doi:10.4178/epih.e2020062)

Supplementary Material 2. Mortality from carbon monoxide poisoning based on data of the National Statistical Office and the National Police Agency

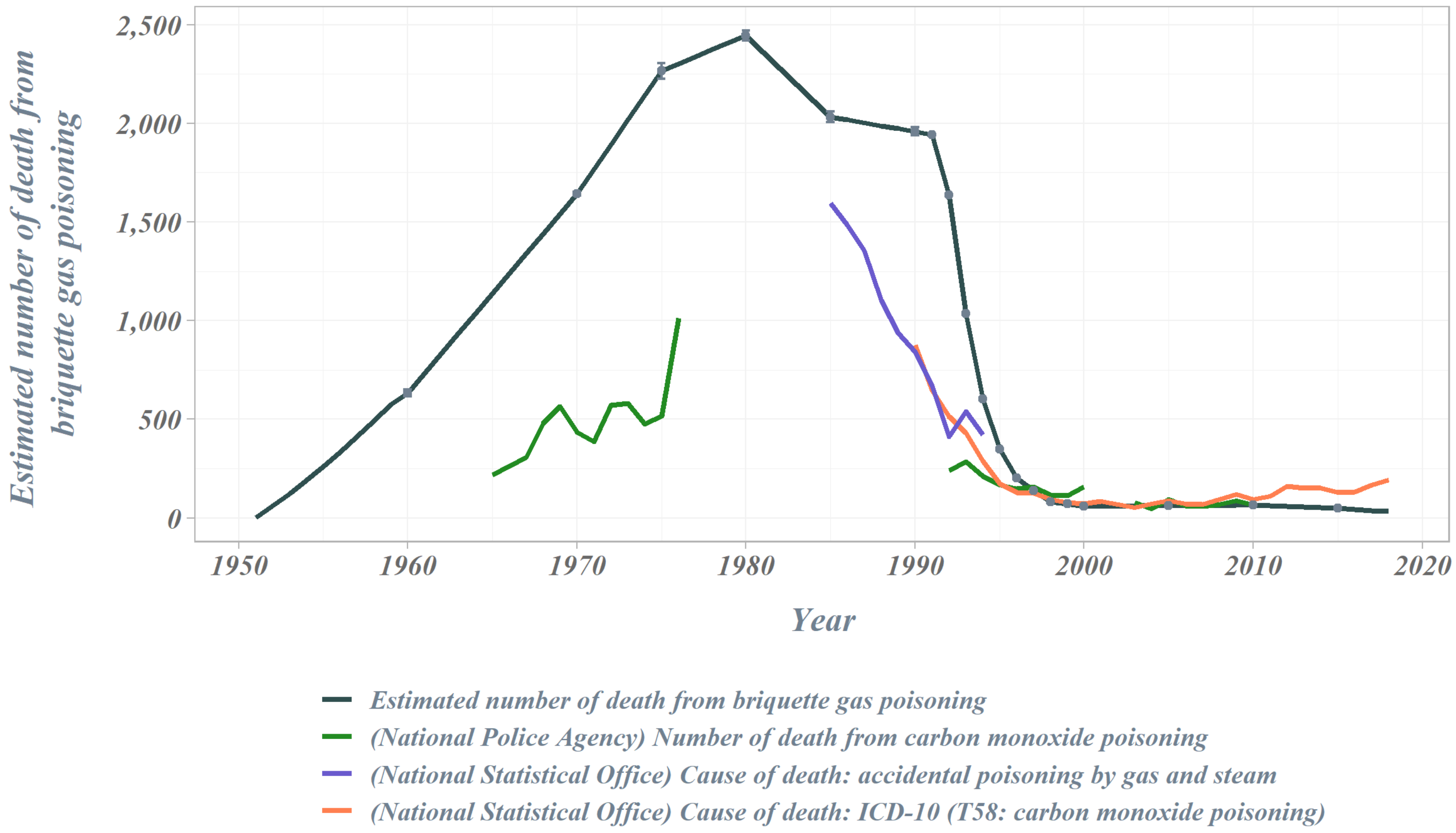

Supplement: Supplementary Material 2. [file epih-42-e2020062-suppl2.pdf]
