# Supplementary material for: Trends of accidental carbon monoxide poisoning in Korea, 1951-2018
Source: Epidemiol Health. 2020 Aug 31;42:e2020062. doi: 10.4178/epih.e2020062 (PMC7871165; doi:10.4178/epih.e2020062)
Supplement: Supplementary Material 3. [file epih-42-e2020062-suppl3.docx]

Supplementary Material 3. Estimated number of victims from carbon dioxide (CO) poisoning, 1951-2018

| Year | Number of households using briquettes as heating fuel | | Number of persons per household | Number of people using  briquette | Number of victims from CO poisoning | | |
| --- | --- | --- | --- | --- | --- | --- | --- |
|  |  |  |  |  | Mild cases | Severe cases | Deaths |
| 1951 | | 3,021 | 5.698 | 17,214 | 649 | 102 | 2 |
| 1952 | | 100,255 | 5.686 | 570,050 | 21,507 | 3,364 | 61 |
| 1953 | | 203,801 | 5.674 | 1,156,367 | 43,628 | 6,825 | 124 |
| 1954 | | 313,657 | 5.662 | 1,775,926 | 67,002 | 10,481 | 191 |
| 1955 | | 429,824 | 5.650 | 2,428,506 | 91,623 | 14,333 | 261 |
| 1956 | | 552,302 | 5.638 | 3,113,879 | 117,481 | 18,378 | 334 |
| 1957 | | 681,091 | 5.626 | 3,831,818 | 144,568 | 22,615 | 412 |
| 1958 | | 816,192 | 5.614 | 4,582,102 | 172,874 | 27,043 | 492 |
| 1959 | | 957,603 | 5.602 | 5,364,492 | 202,393 | 31,660 | 576 |
| 1960 | | 1,053,314 | 5.590 | 5,888,025 | 222,212 | 34,761 | 633 |
| 1961 | | 1,213,948 | 5.555 | 6,743,481 | 257,700 | 40,312 | 734 |
| 1962 | | 1,382,545 | 5.520 | 7,631,648 | 293,188 | 45,864 | 835 |
| 1963 | | 1,559,106 | 5.485 | 8,551,696 | 328,676 | 51,415 | 936 |
| 1964 | | 1,743,631 | 5.450 | 9,502,789 | 364,164 | 56,967 | 1,037 |
| 1965 | | 1,936,121 | 5.415 | 10,484,095 | 399,652 | 62,518 | 1,138 |
| 1966 | | 2,136,575 | 5.380 | 11,494,774 | 435,140 | 68,069 | 1,239 |
| 1967 | | 2,344,993 | 5.345 | 12,533,988 | 470,628 | 73,621 | 1,340 |
| 1968 | | 2,561,375 | 5.310 | 13,600,901 | 506,116 | 79,172 | 1,441 |
| 1969 | | 2,785,721 | 5.275 | 14,694,678 | 541,604 | 84,724 | 1,542 |
| 1970 | | 3,018,032 | 5.240 | 15,814,488 | 577,092 | 90,275 | 1,643 |
| 1971 | | 3,287,766 | 5.200 | 17,096,383 | 620,887 | 97,126 | 1,768 |
| 1972 | | 3,568,377 | 5.160 | 18,412,825 | 664,682 | 103,977 | 1,892 |
| 1973 | | 3,859,868 | 5.120 | 19,762,524 | 708,477 | 110,828 | 2,017 |
| 1974 | | 4,162,235 | 5.080 | 21,144,154 | 752,272 | 117,679 | 2,142 |
| 1975 | | 4,333,725 | 5.040 | 21,841,974 | 796,067 | 124,530 | 2,266 |
| 1976 | | 4,681,864 | 4.942 | 23,137,772 | 808,639 | 126,496 | 2,302 |
| 1977 | | 4,891,366 | 4.844 | 23,693,777 | 821,211 | 128,463 | 2,338 |
| 1978 | | 5,103,989 | 4.746 | 24,223,532 | 833,783 | 130,429 | 2,374 |
| 1979 | | 5,319,732 | 4.648 | 24,726,114 | 846,355 | 132,396 | 2,410 |
| 1980 | | 5,570,471 | 4.550 | 25,345,643 | 858,927 | 134,363 | 2,445 |
| 1981 | | 5,731,452 | 4.458 | 25,550,813 | 829,867 | 129,817 | 2,363 |
| 1982 | | 5,922,003 | 4.366 | 25,855,465 | 800,807 | 125,271 | 2,280 |
| 1983 | | 6,110,246 | 4.274 | 26,115,191 | 771,747 | 120,725 | 2,197 |
| 1984 | | 6,296,182 | 4.182 | 26,330,633 | 742,687 | 116,179 | 2,114 |
| 1985 | | 6,479,811 | 4.090 | 26,502,427 | 713,627 | 111,633 | 2,032 |
| 1986 | | 6,679,270 | 4.014 | 26,810,590 | 708,541 | 110,838 | 2,017 |
| 1987 | | 6,871,033 | 3.938 | 27,058,128 | 703,456 | 110,042 | 2,003 |
| 1988 | | 7,055,100 | 3.862 | 27,246,796 | 698,370 | 109,247 | 1,988 |
| 1989 | | 7,163,175 | 3.786 | 27,119,781 | 693,285 | 108,451 | 1,974 |
| 1990 | | 7,176,360 | 3.710 | 26,624,296 | 688,199 | 107,656 | 1,959 |
| 1991 | | 7,144,000 | 3.636 | 25,975,584 | 681,953 | 106,679 | 1,942 |
| 1992 | | 6,162,000 | 3.562 | 21,949,044 | 574,529 | 89,874 | 1,636 |
| 1993 | | 3,997,000 | 3.488 | 13,941,536 | 363,839 | 56,916 | 1,036 |
| 1994 | | 2,379,000 | 3.414 | 8,121,906 | 211,328 | 33,058 | 602 |
| 1995 | | 1,412,000 | 3.340 | 4,716,080 | 122,342 | 19,138 | 348 |
| 1996 | | 832,000 | 3.296 | 2,742,272 | 70,924 | 11,095 | 202 |
| 1997 | | 588,000 | 3.252 | 1,912,176 | 49,306 | 7,713 | 140 |
| 1998 | | 353,000 | 3.208 | 1,132,424 | 29,111 | 4,554 | 83 |
| 1999 | | 316,000 | 3.164 | 999,824 | 25,625 | 4,008 | 73 |
| 2000 | | 262,626 | 3.120 | 819,393 | 20,969 | 3,280 | 60 |
| 2001 | | 267,012 | 3.072 | 820,261 | 21,079 | 3,297 | 60 |
| 2002 | | 271,152 | 3.024 | 819,964 | 21,190 | 3,315 | 60 |
| 2003 | | 275,364 | 2.976 | 819,483 | 21,300 | 3,332 | 61 |
| 2004 | | 279,702 | 2.928 | 818,967 | 21,410 | 3,349 | 61 |
| 2005 | | 293,464 | 2.880 | 845,176 | 21,521 | 3,366 | 61 |
| 2006 | | 332,272 | 2.856 | 948,969 | 21,878 | 3,422 | 62 |
| 2007 | | 311,923 | 2.832 | 883,366 | 22,235 | 3,478 | 63 |
| 2008 | | 333,460 | 2.808 | 936,356 | 22,592 | 3,534 | 64 |
| 2009 | | 338,340 | 2.784 | 941,939 | 22,949 | 3,590 | 65 |
| 2010 | | 350,293 | 2.760 | 966,809 | 23,306 | 3,646 | 66 |
| 2011 | | 330,220 | 2.712 | 895,557 | 22,197 | 3,472 | 63 |
| 2012 | | 325,026 | 2.664 | 865,869 | 21,088 | 3,299 | 60 |
| 2013 | | 309,502 | 2.616 | 809,657 | 19,978 | 3,125 | 57 |
| 2014 | | 295,328 | 2.568 | 758,402 | 18,869 | 2,952 | 54 |
| 2015 | | 291,263 | 2.520 | 733,983 | 17,760 | 2,778 | 51 |
| 2016 | | 265,328 | 2.472 | 655,891 | 15,735 | 2,461 | 45 |
| 2017 | | 230,388 | 2.424 | 558,461 | 13,310 | 2,082 | 38 |
| 2018 | | 213,906 | 2.376 | 508,241 | 12,113 | 1,895 | 34 |
