# Supplementary figures and images for: Trends of accidental carbon monoxide poisoning in Korea, 1951-2018
Source: Epidemiol Health. 2020 Aug 31;42:e2020062. doi: 10.4178/epih.e2020062 (PMC7871165; doi:10.4178/epih.e2020062)

*Supplementary Material 4. Description of carbon monoxide poisoning causes, 1990-1999*

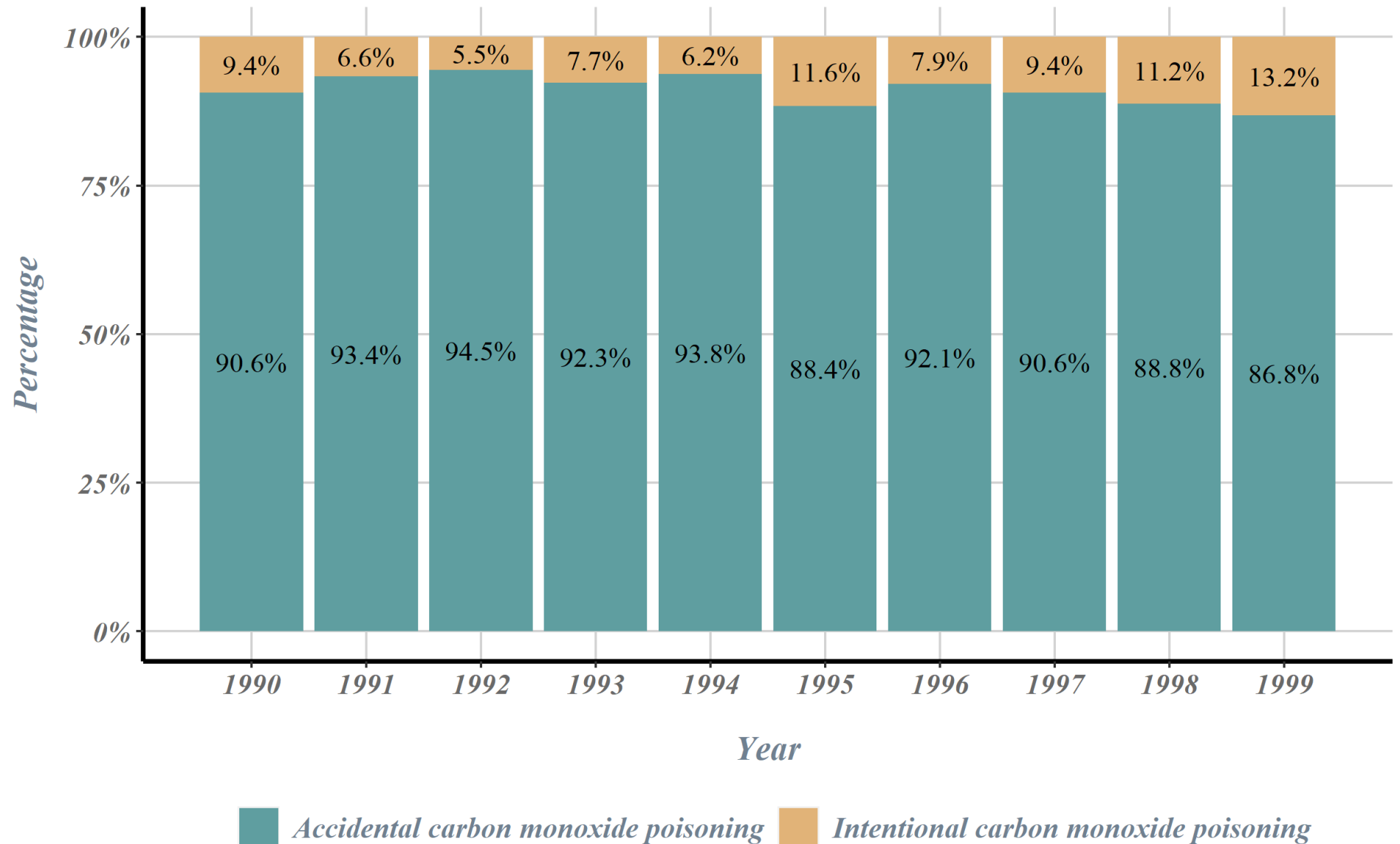

Supplement: Supplementary Material 4. [file epih-42-e2020062-suppl4.pdf]
